# Magnetic Europium Ion-Based Fluorescence Sensing Probes for the Detection of Tetracyclines in Complex Samples

**DOI:** 10.3390/bios16010029

**Published:** 2026-01-01

**Authors:** Miftakhul Jannatin, Yu-Chie Chen

**Affiliations:** 1 Department of Applied Chemistry, National Yang Ming Chiao Tung University, Hsinchu 300, Taiwan; miftakhulj.sc07@nycu.edu.tw; 2 International College of Semiconductor Technology, National Yang Ming Chiao Tung University, Hsinchu 300, Taiwan

**Keywords:** tetracycline, europium ions, magnetic metal ions, fluorescence, magnetic separation, enrichment

## Abstract

Eu^3+^ is a fluorescent and paramagnetic ion whose emission intensity increases when chelated with enhancers such as tetracycline (TC). In this study, Eu^3+^ was conjugated with citric acid (CA) to form magnetic fluorescent probes capable of capturing trace TC from solutions. The probes were rapidly prepared (~2.25 min) and trapped TC within ~2.5 min under microwave heating. The method enabled sensitive detection of TC, oxytetracycline, and chlortetracycline with detection limits of ~3–7 nM by fluorescence spectroscopy. It was successfully applied to real food samples, including fresh chicken broth and commercial broth cubes, achieving high accuracy (93.7% and 96.6%). This dual-functional probe offers a rapid and sensitive approach for detecting TC residues in complex food matrices, demonstrating strong potential for food-safety monitoring.

## 1. Introduction

Antibiotics are vital for managing infections across various fields, such as medicine, animal husbandry, veterinary medicine, aquaculture, and agriculture [1,2,3,4]. However, their extensive use poses significant threats to both the environment and human health [2]. Exposure to antibiotics early in life can lead to increased antimicrobial resistance and disturbances in the developing microbiome, thereby elevating the risk of chronic diseases later [5]. The misuse and overuse of antibiotics have exacerbated the emergence of antimicrobial resistance, a major global health concern [6,7]. Despite their widespread usage, a substantial amount of antibiotics remains unabsorbed by organisms, entering the environment and posing undeniable risks to human life [8]. Tetracyclines (TCs), the second-largest class of antibiotics globally, serve as primary agents for therapeutic purposes, animal disease control, and as major additives in agricultural feed [9] owing to their broad-spectrum effectiveness against various bacteria [10,11]. However, their high water solubility allows them to persist in the environment, contributing to the development and spread of antibiotic-resistant bacterial strains [12,13]. According to the regulations set by the European Union Commission [14] and the Codex Alimentarius Commission [15], the maximum residue limits (MRLs) for TCs are established at 100 μg kg^−1^ and 200 μg kg^−1^, respectively.

To date, the detection of TCs relies predominantly on instruments [16], including high-performance liquid chromatography (HPLC) [10,17,18,19,20], liquid chromatography–mass spectrometry (LC-MS) [9,21,22]. Other analysis tools like enzyme-linked immunosorbent assay (ELISA) [23,24], capillary electrophoresis [25], and electrochemistry [26,27,28,29] are also frequently utilized for TC detection. Although HPLC and LC-MS provide outstanding selectivity and sensitivity, they require costly instrumentation, complex sample pretreatment, trained operators, and large volumes of organic solvents [30,31]. ELISA presents an appealing alternative due to its straightforward operation, rapid response, specificity, and minimal equipment needs [32,33]. However, those above-mentioned methods require costly instruments and sophisticated methods [34].

Conversely, fluorescence-based sensing offers a simple, rapid, low-cost, and highly sensitive approach for on-site TC detection with visual distinguishability [35,36,37,38,39]. For instance, carbon dot-derived materials [35,40,41,42,43], metal–organic framework-based materials [16,44,45,46,47], and aptasensors [48,49,50] have been extensively used as fluorescent sensing probes. Generally, the fabrication time for these sensing probes was rather long [51]. Moreover, sample pretreatment to remove non-target species is crucial for improving selectivity and sensitivity in rapid sensing methods. Magnetic isolation has been widely employed in sample preparation to enrich target analytes while effectively eliminating interfering species [51,52,53,54,55,56]. We previously demonstrated that magnetic metal ions, including Fe^3+^, Ni^2+^, Co^2+^ [51], Gd^3+^ [52,53,54,55], and Eu^3+^ [56], can be directly used as magnetic probes to selectively enrich their target species, such as bacteria, pesticides, and TCs. The magnetic isolation step is essential for removing unbound species/matrix interferences and concentrating the target species, thereby improving sensing signal intensity and reproducibility. This enhancement enables their magnetic isolation using a simple external magnet [52,53,54,55,56]. The trapping affinity between these magnetic ions and the functional groups on the target species can be explained by the Hard and Soft Acid and Base (HSAB) theory [57].

Gd^3+^ has previously been used as a magnetic probe for enriching TCs, followed by Eu^3+^ as a fluorescence-sensing ion [54]. In this study, Eu^3+^ was selected because it uniquely provides both paramagnetism and strong red fluorescence, enabling an integrated magnetic–fluorescence sensing approach. Eu^3+^ possesses six unpaired 4f electrons, imparting paramagnetism, and its emission can be markedly enhanced through the antenna effect upon coordination with the β-diketone moiety of tetracyclines [58]. As a hard Lewis acid, Eu^3+^ readily binds CA and TCs, consistent with HSAB principles [57]. Therefore, Eu^3+^-CA conjugates were employed as dual-function probes capable of magnetic enrichment and fluorescence detection of tetracyclines. Trace amounts of analytes captured on the probes can be quantified based on the enhanced Eu^3+^ emission. Microwave heating [59,60,61,62] was further used to accelerate both probe fabrication and enrichment, reducing the overall analysis time.

## 2. Methods

### 2.1. Materials and Reagents

Europium (III) acetate (99.9%) and oxacillin sodium salt were purchased from Thermo Scientific (Ward Hill, MA, USA). Hydrochloric acid (36.5%), tris(hydroxymethyl)-aminomethane (Tris) base, and Tris hydrochloride were obtained from J. T. Baker (Phillipsburg, NJ, USA). Iron (III) chloride hexahydrate, potassium chloride, and sodium hydroxide were purchased from Honeywell-Fluka (Seelze, Germany). Ampicillin, CA, CAP, dimethyl sulfoxide (DMSO), magnesium chloride, OxTC, sodium acetate anhydrous, TC, and zinc chloride were obtained from Sigma Aldrich (St. Louis, MO, USA). Chlortetracycline hydrochloride (CTC) from *Streptomyces aureofaciens* was purchased from Biochimika-Fluka (Buchs, Switzerland). Calcium chloride dihydrate was obtained from Riedel-de Haën (Seelze, Germany). Sodium chloride was purchased from Ducksan (Ansansi, Gyunggido, Republic of Korea). Pure water used in all the experiments was purchased from Taisun (Taipei City, Taiwan). Neodymium magnets (~4000 Gauss) were purchased from a local store. Chicken broth cubes and chicken meat were obtained from a local shop and a butcher shop, respectively.

### 2.2. Instrumentation

A domestic microwave oven from Shampo (Taipei, Taiwan) was used to accelerate the fabrication of the magnetic probes and the experimental steps of the developed method. All the fluorescence spectra were obtained using a Horiba Fluormax Plus spectrofluorometer (Edison, NJ, USA). The magnetism strength of the as-prepared magnetic conjugates was determined using an MPMS-3 superconducting quantum interference device (SQUID) from American Quantum (San Diego, CA, USA). Transmission electron microscope (TEM) images were obtained from a JEOL JEM-2100 TEM (Tokyo, Japan). An Eyela FDU-1200 freeze dryer (Tokyo, Japan) was used to lyophilize the probes.

### 2.3. Preparation and Optimization of the Magnetic Probes

**Scheme 1:**
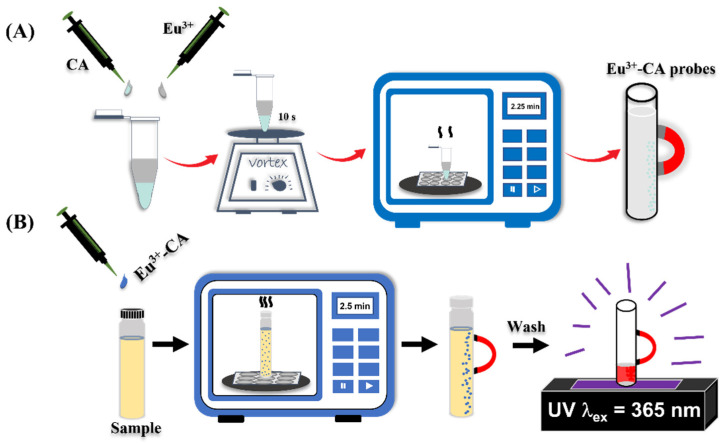
Schematic illustration of (**A**) the preparation of the Eu^3+^-CA probes and (**B**) the sensing steps for the detection of TCs.

The fabrication steps, including incubation methods and compositions of the magnetic probe, were initially optimized. The incubation method of Eu^3+^ and CA was evaluated by comparing vortex-mixing and microwave heating. Aqueous europium acetate (30 mM, 0.2 mL) and CA (20 mM, 0.2 mL) were mixed by vortex-mixing for 10 s, then placed in a water bath (2.5 mL) and subjected to microwave heating (180 W) for 2.25 min (Scheme 1A). In parallel, the same mixture was continuously vortex-mixed 1 h. The resulting suspension underwent magnetic isolation by placing an external magnet (~4000 G) next to the sample until the magnetic isolation was complete, which took ~20 min. The isolated species were rinsed with deionized water (0.35 mL × 2).

Next, to evaluate the optimal molar ratios of Eu^3+^ to CA, europium acetate (30 mM, 0.2 mL) was mixed with CA at different concentrations (60, 30, 15, and 7.5 mM), followed by microwave heating for 2.25 min. Subsequent magnetic separation using an external magnet was conducted until the supernatant became clear. Separation time was 30 min. The isolated conjugates, obtained from the mixtures of Eu^3+^ and CA with different molar ratios, were also subjected to overnight freeze-drying to determine the yield.

### 2.4. Using the Eu^3+^-CA Conjugates as Magnetic Probes Against TC

Magnetic probes were prepared by mixing aqueous europium acetate (30 mM, 0.2 mL) and CA (15 mM, 0.2 mL), followed by vortex-mixing for 10 s, then placed in a water bath (2.5 mL) and subjected to microwave-heating (180 W) for 2.25 min. The resulting suspension underwent magnetic isolation by placing an external magnet (~4000 G) next to the sample for 20 min, followed by rinsing with deionized water (0.35 mL × 2). The magnetically isolated Eu^3+^-CA conjugates (~30 μL) were then used for further experiments. The optimal experimental parameters for the sensing experiments were initially investigated, and the details were described in the additional experimental section in the Supporting Information (SI). The optimal experimental steps were described as follows. The standard solution of TC (10 mM, 0.4 mL) in Tris buffer (pH 6) was prepared and mixed with the Eu^3+^-CA conjugate (15 µL) as obtained above, followed by microwave heating at 180 W for 2.25 min (Scheme 1B). An external magnet (~4000 G) was positioned adjacent to the vial (wall thickness: 0.5 mm; volume: ~0.45 mL) for 20 min to observe the color difference between the blank (Tris buffer only) and the TC-containing system. Similarly, the standard solution (0.4 mL), with and without TC (0.1 mM) in Tris buffer (pH 6), was mixed with the as-prepared magnetic Eu^3+^-CA conjugates (10 µL) in a small glass vial for magnetic isolation for 20 min. At the time point of 20 min, the magnetic isolation was completed based on the naked eye investigation. The resulting conjugates were rinsed twice with Tris buffer (0.35 mL × 2), and the rinsed conjugates (~50 µL) were resuspended in Tris buffer (75 µL, pH 9). The fluorescence spectra of the resuspended samples were then recorded (λ_ex_ = 394 nm).

### 2.5. Effects of Interference Species

Interfering species (400 nM), including K^+^, Na^+^, Ca^2+^, Mg^2+^, Fe^3+^, Zn^2+^, citrate, and acetate, were individually added to the standard solution containing TC (40 nM) to examine their effects. The experimental steps for detecting TC in the as-prepared samples were similar to those described above.

### 2.6. Examination of Selectivity

Non-targeted antibiotics (40 nM), including oxacillin, ampicillin, and CAP, were selected as the models. Ampicillin and CAP were dissolved in DMSO and diluted with Tris (pH 6). The experimental steps were similar to those described above.

### 2.7. Preparation of the Simulated Real Samples

No animal or human subjects were involved in this study. Simulated real samples were prepared by spiking TC into chicken broth, including those prepared from fresh chicken meat and from commercial chicken broth cubes. The freshly prepared chicken broth was prepared by boiling chicken meat (~1.65 g) in deionized water (500 mL) at 100 °C under stirring for 10 min. The resulting broth was filtered with a filter paper (pore size: ~90 μm). The filtrate was 5-fold diluted with deionized water, followed by filtration with a filter membrane (pore size: ~200 nm). The resulting filtrate was 10-fold diluted by Tris Buffer (10 mM, pH 6). The resulting solution (6 mL) was spiked with TC to have the final concentration at 15 nM in the sample. The standard addition method was used to quantify the concentration of TC in the simulated real samples. Thus, the as-prepared simulated sample containing TC was added with additional TC at different concentrations to establish a calibration curve. Three replicates were conducted for each sample with different concentrations.

On the other hand, the chicken broth prepared using chicken broth cubes was prepared by mixing chicken broth cubes (4.38 mg) in deionized water (438 mL) and heating in a water bath at 100 °C under stirring at 300 rpm for 10 min. After reaching room temperature, the solution was filtered twice through standard filter paper and then passed through a 0.2 μm pore-size filter to further remove fine particulates. The resulting filtrate was 10-fold diluted with Tris buffer (pH 6). Additionally, TC (10 nM) was spiked into the as-prepared broth solution. The standard addition method was employed to determine TC in the simulated real samples. Specifically, additional TC at various concentrations was added to the prepared samples. The experimental steps for detecting TC were similar to those described above. Each concentration was tested in triplicate. A calibration curve was established accordingly.

## 3. Results and Discussion

### 3.1. Synthesis and Characterization of the Eu^3+^-CA Conjugates

We initially synthesized the Eu^3+^-CA conjugates using microwave heating, as it is known to accelerate binding processes [59,60,61,62]. SI Figure S1A shows the fluorescence spectra (λ_ex_ = 394 nm) of the samples containing Eu^3+^ alone (black) and the supernatant obtained after incubating Eu^3+^ with CA (blue) under microwave-heating (power: 180 W) for 2.25 min, followed by magnetic isolation. SI Figure S1B shows the fluorescence spectra from the same samples as used to acquire SI Figure S1A, obtained by mixing Eu^3+^ and CA under vortex mixing for 1 h. Notably, the decrease in the fluorescence intensity at 616 nm was more pronounced with microwave-heating (2.25 min) than with vortex-mixing (1 h), indicating that microwave-heating was more effective in accelerating the formation of Eu^3+^-CA conjugates. The temperature of the reaction solution (6 mL) was raised to ~55 °C from room temperature (~25 °C) under microwave-heating with a power of 180 W for 2.25 min. The temperatures of the reaction solution were raised to ~34 and ~76 °C from room temperature when the powers were 90 and 270 W, respectively, under microwave -heating for 2.25 min. 34 °C was a bit low, whereas 76 °C was a bit high for being used to generate the probes. Figure S2A–C show the photographs of the generated probes obtained under microwave-heating with the powers of 90, 180, and 270 W, respectively, followed by magnetic isolation. All the probes could be readily attached to the wall of the vials after 20 min (SI Figure S2). The optical density values at 600 nm of the probes shown in the figure were estimated to be ~0.36, ~0.55, and ~0.81 for samples prepared under microwave heating at powers of 90, 180, and 270 W, respectively. These results indicate that a higher microwave-heating power promotes the formation of a greater amount of conjugates. Nevertheless, a larger amount of precipitates was observed at the bottom of the vial in Figure S2C compared with Figure S2A, suggesting that particles formed under higher microwave-heating power possess larger sizes. Thus, the Eu^3+^-CA conjugates were synthesized under microwave heating with the power of 180 W for 2.25 min in the following studies. The limits of detection (LODs) using the probes obtained from different microwave powers will be discussed later.

Moreover, the optimal composition for forming the Eu^3+^-CA conjugates was examined. By fixing the amount of Eu^3+^, the amount of CA was varied. The supernatant obtained after the formation of Eu^3+^-CA conjugates was subsequently analyzed. Specifically, the fluorescence intensity attributed to free Eu^3+^ decreased as a larger portion of the Eu^3+^–CA conjugates was magnetically separated. SI Figure S3A–D show the fluorescence spectra of the solution containing Eu^3+^ alone (30 mM) (pink) and the solution containing Eu^3+^ (30 mM) and CA (black) at the concentrations of 30 mM, 60 mM, 15 mM, and 7.5 mM, respectively, incubated under microwave-heating followed by magnetic isolation. Evidently, the decrease in the fluorescence intensity at 616 nm was most pronounced in Figure S3C, indicating that the sample containing Eu^3+^ (30 mM) and CA (15 mM) (Eu: CA= 1:0.5) represented the optimal composition. At the concentrations of 30 (SI Figure S3A) and 60 mM (SI Figure S3B) of CA, the fluorescence intensity at the wavelength of 616 nm derived from the solution containing Eu^3+^ alone (pink) was similar to that of the supernatant (black) obtained from the mixture of Eu^3+^ and CA (Figure S3A,B). It appeared that no visible Eu^3+^-CA conjugates were formed when the amount of CA was equal to or twice that of Eu^3+^, as can be seen in the photographs in Figure S4A,B. This is understandable, as the solubility of the Eu^3+^–CA conjugates increased with higher amounts of CA in the mixture (Figure S4). In other words, the relative ratio of Eu^3+^ to CA determines whether visible precipitates derived from the Eu^3+^-CA conjugates can form or not. Only these conjugates can then be isolated by applying an external magnet. When the relative amount of Eu^3+^ to CA was increased, distinct precipitates (circled by the red line) formed by Eu^3+^–CA conjugates were observed (SI Figure S4C,D). The aggregation of paramagnetic ions increases the local magnetic susceptibility and total magnetic moment, enhancing the magnetic force exerted on the visible conjugates and thus facilitating magnetic separation [51,53,56]. Moreover, the yield obtained from the molar ratio of 1:0.5 (Eu^3+^ to CA) was also the highest among the different compositions (SI Figure S4E). Therefore, the mixture of Eu^3+^ and CA with a molar ratio of 1: 0.5 was incubated under microwave-heating for 2.25 min to generate the magnetic probes for the subsequent experiments.

**Figure 1:**
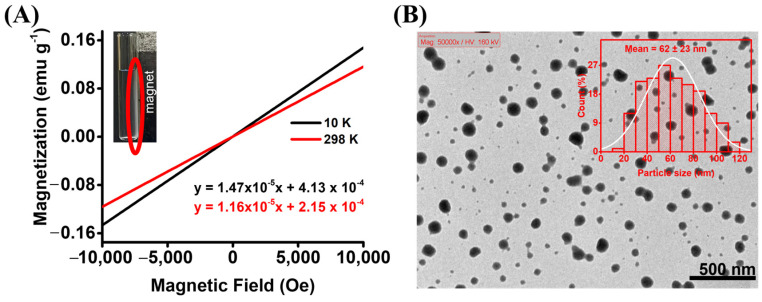
(**A**) Hysteresis curves of the Eu^3+^-CA conjugates obtained at 10 K (black) and 298 K (red), inset: photograph of the magnetic Eu^3+^-CA conjugates with an external magnet next, taken after standing for 20 min. (**B**) TEM image of Eu^3+^-CA conjugates. The inset plot shows the particle size distribution of the Eu^3+^-CA conjugates.

The generated Eu^3+^-CA conjugates exhibited sufficient magnetic responsiveness to be visibly attracted and isolated by an external magnet. That is, the conjugates could be magnetically isolated when applying an external magnet (~4000 Gauss) next to the same vial containing the conjugates (inset photograph in Figure 1A), marked by a red elliptical outline. The magnetic susceptibilities of the Eu^3+^-CA conjugates at 10 and 298 K were estimated to be 1.47 × 10^−5^ and 1.16 × 10^−5^ emu g^−1^, respectively (Figure 1A). The TEM image of these Eu^3+^-TC conjugates revealed that these conjugates were spherical in shape. The inset in Figure 1B shows the particle size distribution, with an average diameter of 62 ± 23 nm (*n* = 172). We then used the as-prepared conjugates as the magnetic and sensing probes for TCs.

### 3.2. Using Magnetic Eu^3+^-CA Conjugates as the Sensing Probe

**Figure 2:**
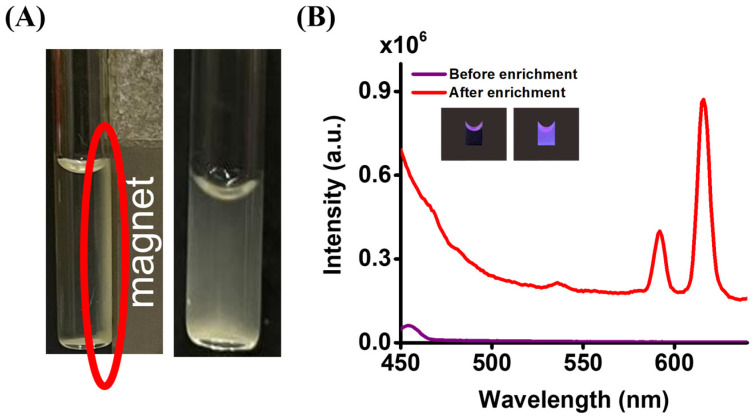
(**A**) Photographs of the samples containing TC (10 mM, 0.4 mL) using the Eu^3+^-CA conjugates (15 µL) as the sensing probes, with (left) and without (right) applying an external magnet. (**B**) Fluorescence spectra of the samples TC (0.1 mM, 0.4 mL) obtained before (purple) and after (red) the addition of 10 µL probes, followed by magnetic separation (λ_ex_ = 394 nm). The inset shows photographs of the corresponding samples captured under UV illumination (λ_max_ = 365 nm).

We employed the as-prepared magnetic probes for detecting TC. To make the change visible to the naked eye, a standard solution containing TC at a relatively high concentration, which appeared yellowish, was used as the model sample. Figure 2A shows the photographs of the standard solution containing TC (10 mM) and incubated with the Eu^3+^-CA conjugates, taken after removing the supernatant, followed by the rinse step and re-suspension in a small volume of Tris buffer, with (left) and without (right) the application of an external magnet. It was noteworthy that the supernatant in the vial on the left (Figure 2A) became colorless after magnetic isolation, while the magnetic conjugates exhibited a yellowish appearance. These observations indicated that TC was successfully captured by the magnetic probes. Figure 2B presents the fluorescence spectra of the samples containing TC at a low concentration (0.1 mM) obtained before (purple) and after (red) the addition of the probes, followed by magnetic separation. The inset photographs illustrate the appearance of the samples under UV light irradiation. No noticeable fluorescence was observed before enrichment (inset photograph in Figure 2B, left). In contrast, bright fluorescence appeared in the vial after magnetic enrichment using the Eu^3+^-TC probes (inset photograph in Figure 2B, right). The fluorescence enhancement of Eu^3+^ was attributed to two factors: the Eu^3+^–CA probes enriched trace amounts of TC in the sample solution, and the binding of TC to the probes further amplified the fluorescence derived from Eu^3+^ via the antenna effect [52]. The β-diketone moiety in TC shows strong coordination with Eu^3+^ [63]. That is, TC absorbs light energy and transfers it to the metal center, resulting in strong luminescence from Eu^3+^ [46].

**Figure 3:**
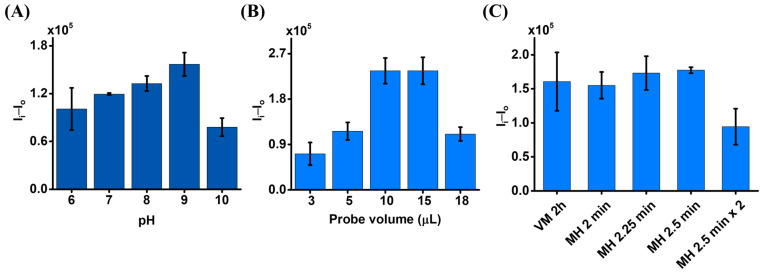
Examination of the optimal experimental parameters, Bar graphs obtained from examining (**A**) pH, (**B**) the volume of the Eu^3+^-CA conjugates, and (**C**) the incubation method for the fabrication of the probes. A standard solution containing TC (40 nM) was used as the test sample.

The optimal experimental parameters were examined (Figure 3). It is reasonable that pH 9 was identified as the optimal condition (Figure 3A), as pH values above 9 can cause hydroxylation of Eu^3+^, thereby diminishing its ability to effectively bind with TC. Furthermore, increasing the amount of Eu^3+^-CA probes resulted in a higher fluorescence background, leading to reduced detection sensitivity for TC. In addition, since the results obtained from vortex mixing for 1 h and microwave heating for 2.5 min were comparable, the shorter incubation time of 2.5 min was selected for subsequent experiments. Thus, the optimal parameters, i.e., pH 9, 10 μL of probe, and microwave heating for 2.5 min, were employed in the subsequent studies.

**Figure 4:**
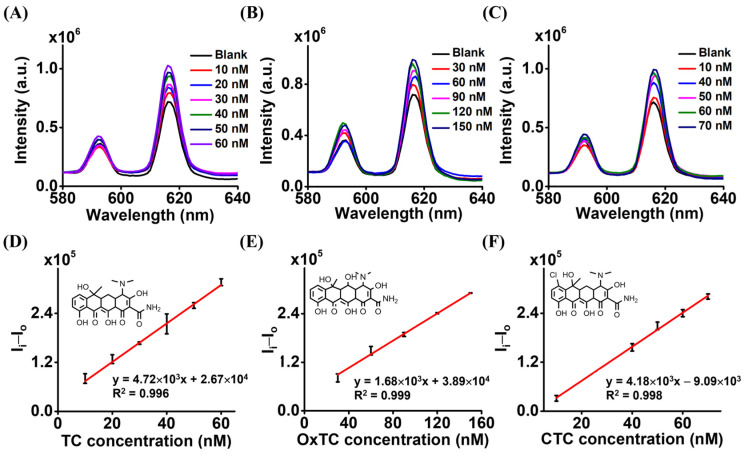
Examination of quantitative analysis. Representative fluorescence spectra of the samples (6 mL) containing (**A**) TC, (**B**) oxTC, and (**C**) CTC at different concentrations obtained using our enrichment and sensing method with the Eu^3+^-CA conjugates as the probes and their corresponding plots (**D**–**F**) based on three replicates. I_i_ and I_0_ stand for the fluorescence intensity at 616 nm derived from the sample and the blank spectra, respectively.

Since the β-diketone structure is common among TC analogs, it is likely that other TCs, such as oxTC and CTC, can also be detected using the optimized parameters as summarized above. Thus, in addition to TC, OxTC, and CTC at various concentrations were used as model analytes to evaluate the feasibility of using the developed method as the enrichment and sensing method for quantitative analysis. Figure 4A–C show the representative fluorescence spectra of the samples containing TC, oxTC, and CTC, respectively, at different concentrations obtained after being treated by our method. Figure 4D–F display the corresponding plot, obtained by plotting the difference in the fluorescence intensity at 616 nm between the sample (I_i_) and the blank control (I_0_) versus the concentration of the analyte. Accordingly, the sensitivity of our method toward TC and CTC was comparable, as indicated by the similar slopes of their regression equations. The LODs for TC, oxTC, and CTC were determined to be 3.1, 7.3, and 3.6 nM, respectively, based on the 3σ/slope method, where σ represents the standard deviation obtained from three replicate measurements of the blank fluorescence intensity at 616 nm. These LOD values are notably lower than the MRLs established by the European Union (100 nM in feed) [14] and the Food and Agriculture Organization of the United Nations (100 nM in milk) [15].

Moreover, one might wonder the LODs achieved using probes generated under microwave heating at powers of 90 and 270 W, in addition to the power of 180 W stated above. Figure S5A,B display the fluorescence spectra recorded at different TC concentrations, while Figure S5C,D present the corresponding calibration plots. The LODs, calculated as 3σ/S, obtained using probes prepared at 90 and 270 W were ~14 and ~16 nM, respectively, both of which are inferior to the LOD achieved using probes prepared at 180 W. The poorer performance observed for probes prepared at 90 W may be attributed to insufficient probe robustness during sample preparation, whereas the probes generated at 270 W are likely larger in size, which may adversely affect magnetic separation and affinity efficiency.

### 3.3. Evaluation of Precision and Accuracy of the Developed Method

To evaluate the precision and accuracy of the developed method, a sample containing TC (40 nM) was used as the model sample. The same samples were examined six times per day over a period of five days. The concentration for each measurement was determined using the calibration curve (y = 4.72 × 10^3^ x + 2.67 × 10^3^, R^2^ = 0.996) provided in Figure 4D. Figure S6A,B show the resulting fluorescence spectra obtained from the samples containing TC and the blanks containing Tris buffer, respectively. Figure S6C shows the summarized bar graphs. Table S1 lists the calculated concentrations of TC from those 30 runs. Accordingly, the average concentration from 30 runs was 40.4 ± 1.6 nM. The precision was 3.9%, and the accuracy was ~99%. These results indicated that the developed method possesses high accuracy and desirable reproducibility.

### 3.4. Examination of Interference Effects and Selectivity

**Figure 5:**
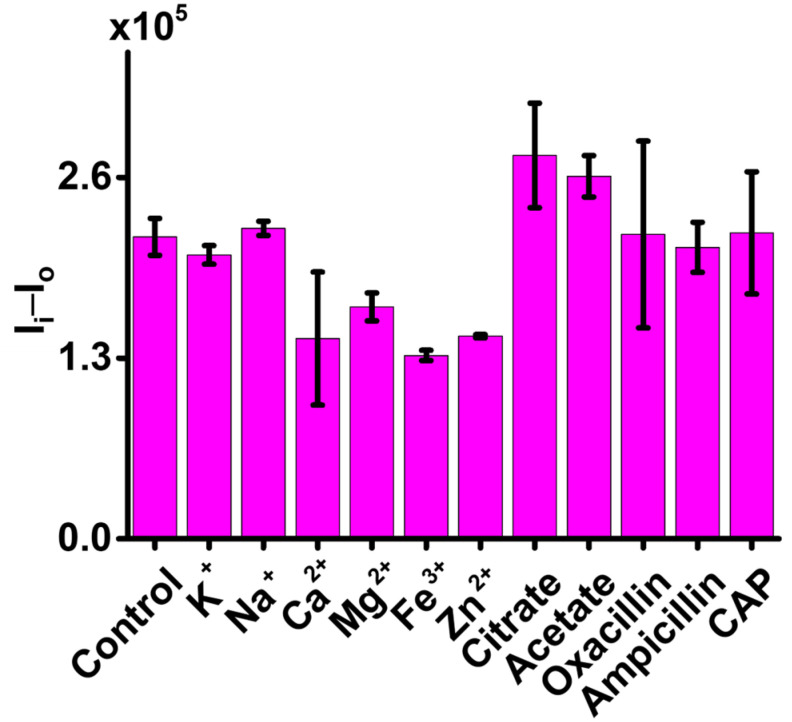
Examination of interference effects and selectivity. Bar graphs obtained by summarizing the results from Figure S7. I_i_ and I_0_ stand for the fluorescence intensity at 616 nm derived from the sample and the blank spectra, respectively.

We further examined the interference effects. Metal ions, including K^+^, Na^+^, Ca^2+^, Mg^2+^, Fe^3+^, Zn^2+^, and anionic ions, including citrate and acetate, commonly found in food samples, were selected as the interference species. Moreover, antibiotics, including oxacillin, ampicillin, and CAP, were used as the models to examine the selectivity of the developed method toward TC. That is, we prepared the samples containing TC (40 nM) spiked with different model interferences or model antibiotics at a concentration of 400 nM. Control stands for the sample only containing TC (40 nM). Figure S7 shows the resulting fluorescence spectra obtained using our method for the detection of TC, whereas Figure 5 shows the bar graphs of the results summarized from Figure S7. The presence of Na^+^ and K^+^ did not significantly affect the detection of TC. According to the HSAB theory, TCs mainly provide hard-base oxygen donors such as β-diketone and phenolate groups, favoring coordination with hard Lewis acids. Eu^3+^, a hard oxophilic cation, forms inner-sphere Eu^3+^–TC chelates that enhance fluorescence by replacing coordinated water through ligand-to-metal energy transfer. Fe^3+^, an even harder acid, competes strongly for the same donor sites, forming non-emissive Fe^3+^-TC complexes that reduce Eu^3+^ fluorescence intensity. In contrast, Na^+^ and K^+^, though hard, are weakly complexing and interact primarily via outer-sphere electrostatics, resulting in negligible interference. In addition, the addition of Ca^2+^, Mg^2+^, and Zn^2+^ in the samples caused a decrease in the fluorescence. Presumably, those cations competed with Eu^3+^ to bind with TC, leading to the reduction in the fluorescence intensity. Conversely, the presence of anions, including citrate and acetate, could induce the additional Eu^3+^ conjugate formation during magnetic separation, leading to the enhancement of the fluorescence derived from Eu^3+^. Citrate also helps moderate Fe^3+^ competition by preferentially binding Fe^3+^, thereby sustaining Eu^3+^-TC complexation and improving fluorescence stability. Moreover, the presence of other non-target antibiotics, including oxacillin, ampicillin, and CAP, had a fluorescence intensity similar to that of the control sample, i.e., the sample containing TC only. That is, the developed method possesses good selectivity toward TC.

### 3.5. Analysis of Simulated Real Samples

**Figure 6:**
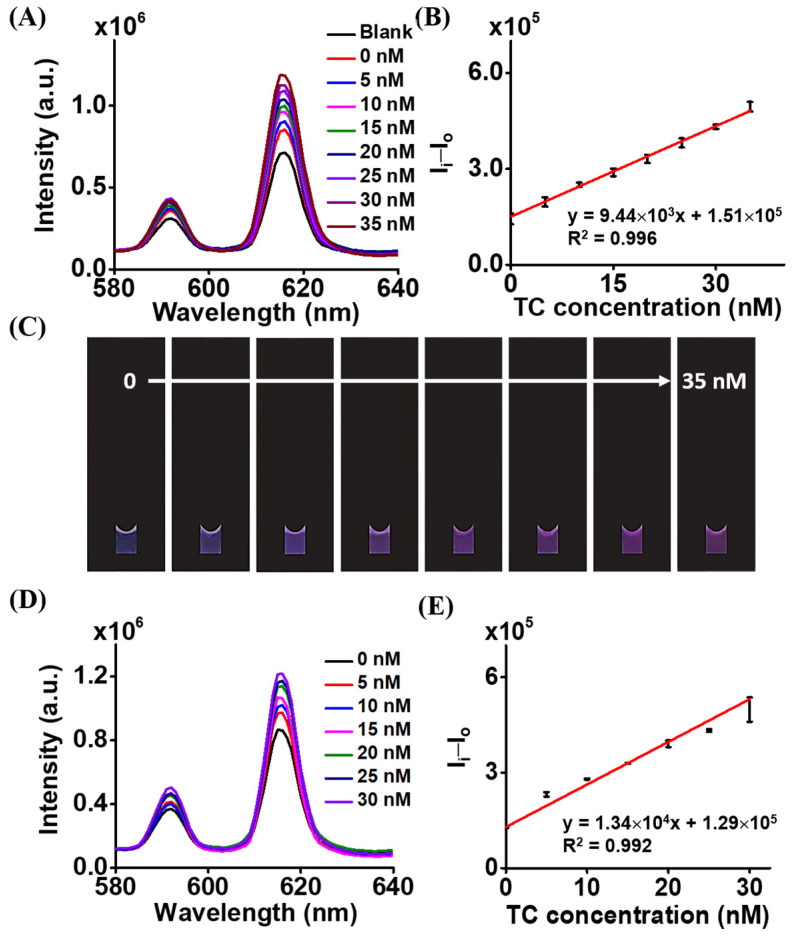
Quantitative analysis of the simulated real samples. (**A**) Representative fluorescence spectra and (**B**) the corresponding calibration curve obtained from the broth prepared using fresh chicken meat, spiked with TC (15 nM), and analyzed using the developed method. (**C**) Photographs of the corresponding samples used in panels (**A**,**B**), taken under UV light irradiation (λ_max_ = 365 nm). (**D**) Representative fluorescence spectra and (**E**) the corresponding calibration curve obtained from the broth prepared using commercial chicken broth cubes, spiked with TC (10 nM), and analyzed using the developed method.

To evaluate the feasibility of applying the developed method for TC detection in real-world samples, TC was spiked into chicken broth prepared from fresh chicken meat and commercial chicken broth cubes. The standard addition method was employed to determine the TC concentration in the prepared samples. Specifically, varying concentrations of TC were added to the broth sample that had been previously spiked with TC. Figure 6A shows the resulting fluorescence spectra of samples prepared from fresh chicken broth containing TC (15 nM), further spiked with varying concentrations of TC, and analyzed using the developed method. Figure 6B presents the corresponding plot of I_i_-I_0_ versus the added TC concentrations. I_i_ and I_0_ stand for TC after mixing with Eu^3+^-CA conjugates and Tris buffer (pH 9) after mixing with Eu^3+^-CA conjugates, respectively. The concentration of TC in the sample was determined to be ~15.9 nM, indicating ~93.7% accuracy. Figure 6C shows photographs of the samples spiked with additional TC, taken after analysis using the developed method. Apparently, the luminescence became brighter with increasing concentrations of spiked TC. Figure 6D shows the fluorescence spectra of samples prepared from chicken broth cubes spiked with TC (10 nM), followed by the addition of varying TC concentrations for standard addition analysis using the developed method to determine TC levels in the sample. Figure 6E presents the corresponding calibration curve based on the data from Figure 6C. The TC concentration in the sample was determined to be 9.63 nM, indicating an accuracy of ~96.6%. These results demonstrated the feasibility of using the developed method for the detection of TC in real-world samples. The signal-to-noise ratio (S/N) values obtained for chicken-cube broth spiked with 10 nM TC and the broth derived from fresh chicken meat spiked with 15 nM TC were approximately 115 and 198, respectively. These relatively high S/N values indicate that, although the food matrices are complex, matrix-derived interference does not significantly deteriorate the LODs of the proposed method within the tested concentration range.

In addition, chicken broth samples were spiked with higher concentrations of TC (50, 100, and 200 μg kg^−1^ corresponding to 112.5, 225, and 450 nM, respectively) to further evaluate the feasibility of the proposed method for samples containing different TC levels (SI Figure S8). The measured TC concentrations in the spiked chicken broth were approximately 44, 82, and 151 μg kg^−1^ for the nominal concentrations of 50, 100, and 200 μg kg^−1^, respectively. The corresponding relative standard deviations (RSDs) were 2.6%, 6.6%, and 2.6%, indicating good analytical precision. Nevertheless, the accuracy of the measured values declined as the concentration of TC spiked in the chicken broth increased. This decrease in accuracy is presumably attributable to the TC concentration exceeding the optimal working range of the method. Under such conditions, the method parameters used in Figure 6 for these samples in SI Figure S8 may require adjustment to accommodate samples containing higher TC concentrations (e.g., 100 nM).

### 3.6. Blind Sample Test

Because authentic samples containing endogenous TCs were not available, blind samples were prepared in-house by spiking known concentrations of TCs into the sample matrix, with the concentrations known only to the sample preparers. Twelve samples were analyzed by one of the co-authors without prior knowledge of their identities. Figure S9 displays the resulting fluorescence spectra of the twelve samples obtained using our method. As summarized in Table S2, 10 of the 12 samples were correctly classified as TC-positive or TC-negative, while two samples yielded false-positive results. The corresponding false-positive rate was approximately 17%. These results demonstrate the feasibility of the developed method for rapid TC screening in unknown samples, while also suggesting the need for confirmatory analysis or further optimization to reduce the observed false-positive rate.

### 3.7. Comparison of the Developed Method with the Existing Methods

We also compared the developed method with the existing methods [50,64,65,66,67,68,69,70,71,72,73,74,75] from the literature (Table S3). Our method offers a lower LOD and higher sensitivity than most existing methods, especially those probes containing Eu^3+^ [64,65,66]. Moreover, a key advantage of our method over existing approaches is that the probes possess magnetic properties, enabling efficient enrichment of trace amounts of TC. Furthermore, the preparation of our probes is rapid (~2 min). This is significantly shorter than that of the existing methods, which often require lengthy probe synthesis (Table S3).

## 4. Conclusions

Eu^3+^-based sensing methods have been widely applied for the detection of TC due to their good binding selectivity and the antenna effect. Existing approaches primarily focus on the fluorescence properties of Eu^3+^. In our previous study [56], free Eu^3+^ ions were added directly to aqueous samples and exhibited no appreciable magnetism; magnetic isolation occurred only after Eu^3+^ associated with bacterial surfaces. In contrast, the present work developed a fundamentally different probe design by first coordinating Eu^3+^ with CA to form Eu^3+^-CA complexes exhibiting intrinsically enhanced magnetic properties. These Eu^3+^-CA probes can be readily isolated by an external magnet and efficiently enrich TCs before fluorescence detection. This strengthened magnetism arising from Eu^3+^–CA conjugation, rather than free Eu^3+^ ions, represents the key novelty distinguishing this study from our earlier report. Additionally, binding of TCs to the magnetic probes further enhances the Eu^3+^ fluorescence, eliminating the need for any external fluorescence enhancers. To the best of our knowledge, this is the first report that employs both the fluorescence and paramagnetic properties of Eu^3+^ for the enrichment and detection of TCs in complex samples. As a result, our Eu^3+^-CA probes function not only as fluorescent sensors but also as magnetic probes. These dual functionalities contribute to the enhanced sensitivity of the method, yielding a low LOD for TCs. A key advantage of the developed method is the short fabrication time of the Eu^3+^-CA probes. Nevertheless, the sensor response may degrade if the probes are stored for extended periods due to potential changes in the coordination structure. Future work may explore strategies to enhance stability for applications that require extended probe lifetimes. However, the cost of one probe is low, e.g., $~0.17. Beyond its analytical strengths, the method holds important practical implications. The growing concern over antibiotic residues in food, especially TCs in meat and broth products, demands fast, reliable, and field-deployable detection tools. Our approach addresses this need by offering a portable, low-cost, and time-efficient strategy for on-site screening. The integration of this platform with miniaturized fluorescence readers could further expand its applicability for point-of-care diagnostics and environmental monitoring. The ability of the developed method to rapidly screen for TC offers new opportunities for fast and efficient detection, an essential feature for ensuring food safety in light of the serious risks associated with antibiotic contamination. In addition, beyond conventional benchtop fluorescence measurements, recent advances in micro- and nano-scale optical devices, such as high-speed multiwavelength micro-LEDs and dynamically reconfigurable graphene metasurfaces, highlight emerging opportunities for next-generation optical sensing platforms [76,77]. Although these technologies have not yet been applied to TC analysis, their future integration with selective fluorescence probes, including the magnetic europium ion-based system developed in this work, may enable sensing devices with improved miniaturization, adaptability, and functionality.

## Data Availability

Data are available upon request.

## Supplementary Materials

The following supporting information can be downloaded at: https://www.mdpi.com/article/10.3390/bios16010029/s1.
