# Supplementary material for: Magnetic Europium Ion-Based Fluorescence Sensing Probes for the Detection of Tetracyclines in Complex Samples
Source: Biosensors (Basel). 2026 Jan 1;16(1):29. doi: 10.3390/bios16010029 (PMC12839073; doi:10.3390/bios16010029)
Supplement: Supplementary file 1 [file biosensors-16-00029-s001.zip › biosensors-4015425-supplementary.pdf]

biosensors

MDPI

Supporting Information

Magnetic Europium Ion-Based Fluorescence Sensing Probes for the Detection of Tetracyclines in Complex Samples

Miftakhul Jannatin [1] and Yu-Chie Chen [1,2,*]

[1] Department of Applied Chemistry, National Yang Ming Chiao Tung University, Hsinchu 300, Taiwan; miftakhulj.sc07@nycu.edu.tw
[2] International College of Semiconductor Technology, National Yang Ming Chiao Tung University, Hsinchu 300, Taiwan
* Correspondence: yuchie@nycu.edu.tw; Tel: +886-3-5131527; Fax: +886-3-5723764

Additional Experimental Section

Optimization of the experimental parameters in the sensing experiment

Optimization of key experimental parameters, including pH, probe volume, and incubation method, was carried out to establish the optimal conditions for the developed method. The experiments were performed by mixing a standard solution of tetracycline (TC) (40 nM) with the Eu^{3+}-CA conjugate under various conditions. TC stock solutions were prepared in methanol and subsequently diluted with Tris buffer to the desired concentrations during sample preparation. Tris buffer (10 mM) with different pH values (6, 7, 8, 9, and 10) was used as the solvent for TC prior to mixing with 10 μL of the probe, followed by microwave heating at 180 W for 2.25 min. An external magnet (~4000 G) was placed next to the resulting sample vial for 20 min. 20 min was determined because the magnetic conjugates were nearly completed based on the naked eye investigation. The supernatant (5.6 mL) was removed, and the remaining conjugates (0.4 mL) were moved to a small glass vial (wall thickness: 0.5 mm, vial volume: ~0.45 mL) for further magnetic isolation for 20 min. The resulting conjugates were rinsed with the same Tris buffer (0.35 mL × 2). The rinsed conjugates (~50 μL) were then resuspended in Tris buffer (75 μL, pH 9), followed by examination with fluorescence spectroscopy (λ_{ex}= 394 nm). The optimal pH obtained from these experiments was subsequently employed to investigate the effect of probe volumes (3, 5, 10, 15, and 18 μL) under the same experimental conditions as used in the previous optimization. The optimized pH and probe volume were subsequently employed to refine the incubation conditions. Comparative experiments were conducted between conventional vortex mixing for 2 h and microwave-assisted incubation at 180 W for 2.0, 2.25, 2.5, and two successive 2.5 min cycles. Magnetic isolation and rinsing were carried out as optimized in the preceding experiments. To evaluate the analytical performance, the prepared Eu^{3+}-CA conjugate (10 μL) was added to 6 mL of sample solution containing various concentrations of TC analogs (TC, oxTC, or CTC) in Tris buffer (10 mM, pH 6). The mixture was vortexed for 10 s, placed in a 2.5 mL water bath, and subsequently subjected to microwave irradiation at 180 W for 2.5 min.

Preparation of the samples spiked with TC at different concentrations.

Chicken broth was first prepared as the matrix solution. Specifically, 9.63 mg of chicken broth powder was dissolved in 482 mL of deionized water to obtain a concentration of 0.02 mg mL^{-1}. This solution was further diluted twofold with deionized water to yield a concentration of 0.01 mg mL^{-1}, followed by a tenfold dilution using Tris buffer (pH 6.0) by adding 20 mL of the broth solution, bringing the total volume to 200 mL. The

 https://doi.org/10.3390/bios16010029

resulting solution was used as the solvent for subsequent experiments and was designated as the "No TC" sample in the fluorescence spectra. For TC preparation, 2.59 mg of TC was dissolved in 1 mL of methanol to obtain a stock solution with a concentration of 2.59×10^6 μg kg^{-1}. This stock solution was diluted 100-fold by adding 10 μL to 990 μL of Tris buffer (pH 6.0), yielding a concentration of 2.59×10^4 μg kg^{-1}, and then further diluted 5.18-fold by mixing 386 μL of the solution with 1614 μL of Tris buffer (pH 6.0) to obtain a working solution of 5000 μg kg^{-1}. TC-spiked samples were prepared by adding 1.2, 2.4, and 4.8 mL of the working TC solution to 118.8, 117.6, and 115.2 mL of Tris buffer (pH 6.0), respectively, to achieve final TC concentrations of 50, 100, and 200 μg kg^{-1}. This solution was used as the TC solvent and designated as the 0 μg kg^{-1} TC control. For the standard addition method, TC was further added to the TC-spiked solutions to obtain final concentrations of 30-150 μg kg^{-1} for the 50 and 100 μg kg^{-1} spiked samples and 10-320 μg kg^{-1} for the 200 μg kg^{-1} spiked sample.

Table S1. Evaluation of the accuracy and precision of the developed method based on repeated analysis of a sample containing 40 nM TC, performed six times per day over five consecutive days.

	Morning			Afternoon			Mean	%RSD
**Day 1**	40.6	42.1	42.0	42.0	41.8	41.9	41.7	1.4
**Day 2**	41.8	41.3	41.3	41.2	40.3	40.2	41.0	1.5
**Day 3**	39.9	40.5	40.5	40.1	39.0	36.3	39.4	4.1
**Day 4**	36.4	36.1	41.1	41.2	40.7	40.7	39.3	6.2
**Day 5**	40.6	40.1	40.5	40.5	40.5	40.6	40.5	0.5
Mean = 40.4 ± 1.6 (nM)			%RSD= 3.9%			Accuracy= 99.0%		

Table S2. List of the results from the blind test.

No	Spiked concentration (nM)	Detected
A	10	Yes
B	0	No
C	0	No
D	10	Yes
E	0	No
F	0	No
G	10	Yes
H	0	Yes
I	20	Yes
J	30	Yes
K	0	Yes
L	40	Yes

https://doi.org/10.3390/bios16010029

Table S3. Comparison of our method with the existing methods.

**Fluorescence sensing probes**	**LOD**	**Magnetic enrichment**	**Probe synthesis time**	**Analysis required time**	**Real Sample**	**Ref**
Carbon nitride nanoparticles/CN NPs	120 nM	No	84 h	30 min	Frozen shrimp and river water	[1]
MoS_2 QDs and Eu^{3+}	2 nM	No	44 h	1min	Mouse serum and water sample	[2]
Liposome-encapsulated TPE + Eu^{3+}	28.83 nM	No	>12 h	2 min	Milk, lake & tap water	[3]
FITC + Eu^{3+}	7.1 nM	No	>61 h	10 min	Milk	[4]
Fluorescent Brightener KS-N + Eu^{3+}	17.9 nM	No	>60 h	1 min	Milk & honey	[5]
g-C_3N_4 + Eu^{3+}	6.5 nM	No	>26 h	10 min	Milk	[6]
C-g-C_3N_4 + Eu^{3+}	7.7 nM	No	>49 h	15 min	Tap water	[7]
CDs + Eu^{3+}	8.7 nM	No	> 82 h	5 min	Milk, honey, lake & tap water	[8]
SiQDs + Eu^{3+}	7.1 nM	No	>102 h	1 min	Milk, honey, lake & tap water	[9]
Luminol + Eu^{3+}	39 nM	No	6 h	10 min	Milk, honey, river & tap water	[10]
DPA-Ce-GMP + Eu^{3+}	6.6 nM	No	>12 h	2 min	Milk	[11]
Laponite + Eu^{3+}	9.5 nM	No	29 h	1 min	Milk, honey & lake water	[12]
Gd0.9@Eu0.1	14 nM	No	96 h	2min	Pork	[13]
Eu^{3+}-CA	~3 nM	Yes	2.25 min	2.5 min	Broth from fresh chicken meat and cubes	This work

https://doi.org/10.3390/bios16010029

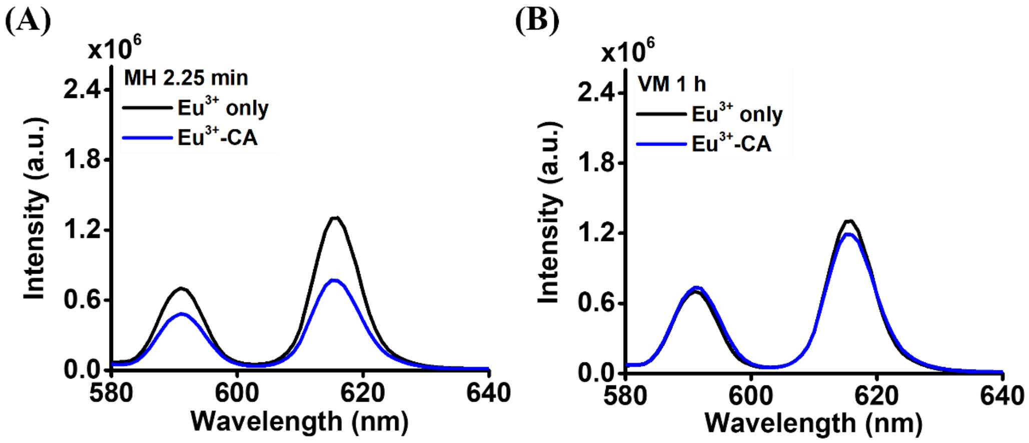

Figure S1. Examination of the methods for preparing the Eu^{3+}-CA conjugates. Representative fluorescence spectra (λ_{ex}= 394 nm) of the supernatants of the samples (0.2 mL) containing Eu^{3+} (0.03 M) obtained from individually incubated with aqueous CA (0.02 M, 0.2 mL) (blue) and water (black) under (A) microwave-heating (MH) (power: 180 W) for 2.25 min and (B) vortex-mixing (VM) for 1 h, followed by magnetic isolation.

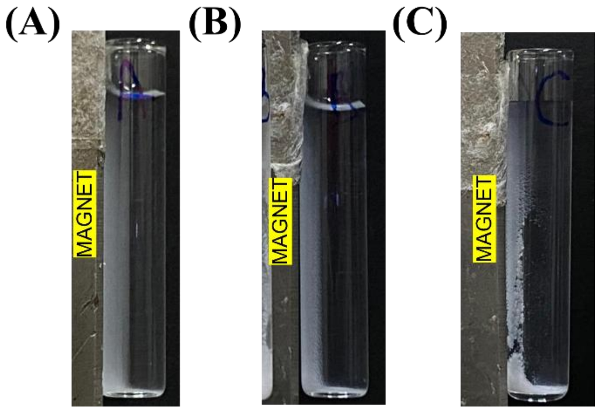

Figure S2. Photographs of the generated probes obtained after heating in a microwave oven with the powers of (A) 90, (B) 180, and (C) 270 W, followed by magnetic isolation for 20 min.

https://doi.org/10.3390/bios16010029

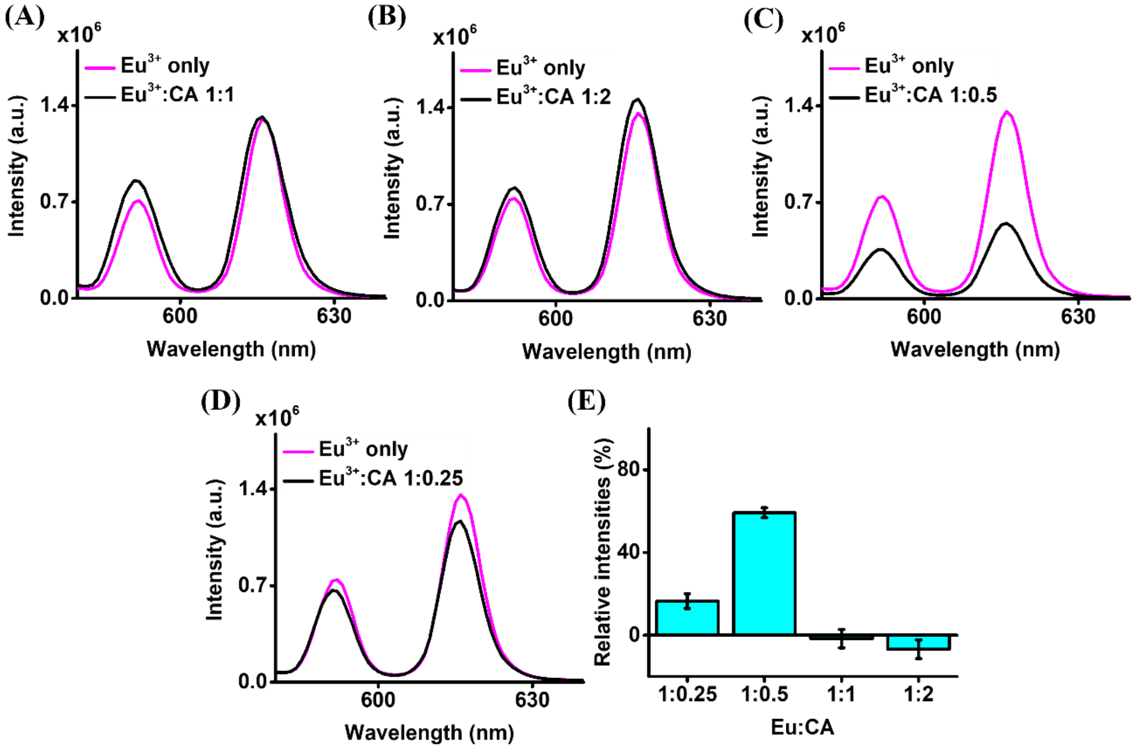

Figure S3. Examination of the optimal molar ratio of Eu^{3+} to CA. Representative fluorescence spectra (λ_{ex}= 394 nm) of the supernatants of the solutions (0.2 mL) containing Eu^{3+} (30 mM) without (pink) and with individual incubation with CA (black) at the concentrations of (A) 30 mM, (B) 60 mM, (C) 15 mM, and (D) 7.5 mM and water (purple) under MH (power: 180 W) for 2.25 min, followed by magnetic isolation. Three replicates were conducted. (E) The summarized bar graphs of the results obtained from Panels (A)-(D) with three replicates. I_0 and I_i stand for the fluorescence intensity at 616 nm derived from the purple and black spectra, respectively.

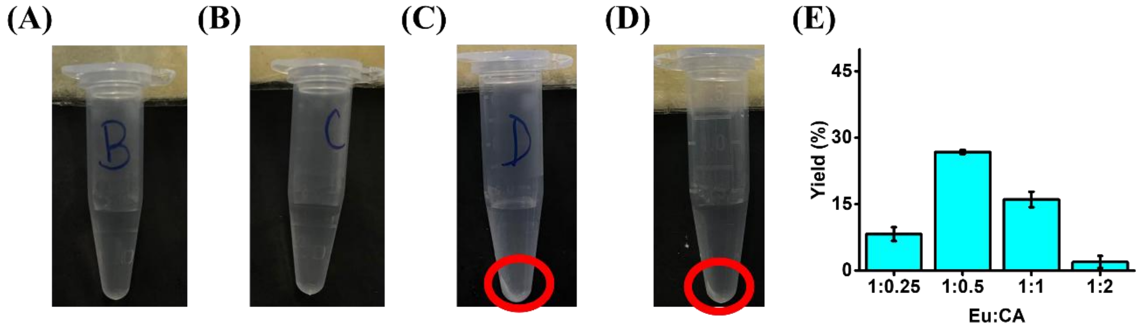

Figure S4. Photographs of the samples containing Eu^{3+} (0.2 mL) after incubation with CA at molar ratios of (A) 1:2, (B) 1:1, (C) 1:0.5, and (D) 1:0.25 (Eu^{3+} to CA) under microwave heating (180 W) for 2.25 min, followed by centrifugation at (6,000 rpm, 10 min). (E) Bar graphs summarizing the yield% obtained from the combinations of Eu^{3+} and CA with different molar ratios.

https://doi.org/10.3390/bios16010029

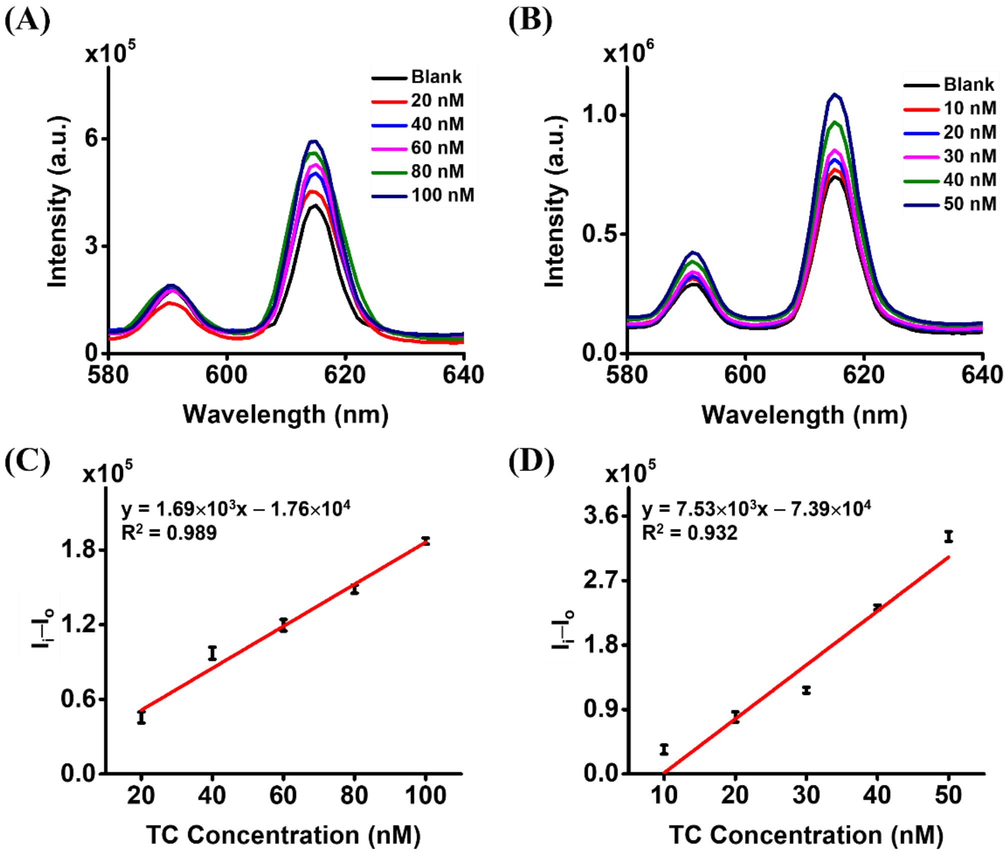

Figure S5. Examination of the LODs by using the probes generated from different microwave powers. Representative fluorescence spectra of the samples (6 mL) containing TC at different concentrations obtained using the Eu^{3+}-CA conjugates as the probes, which were generated under microwave-heating with the powers of (A) 90 and (B) 270 W and their corresponding calibration plots derived from the powers of (C) 90 W (C) and (D) 270 W based on three replicates. I_i and I_0 stand for the fluorescence intensities at 616 nm derived from the sample and the blank spectra, respectively.

https://doi.org/10.3390/bios16010029

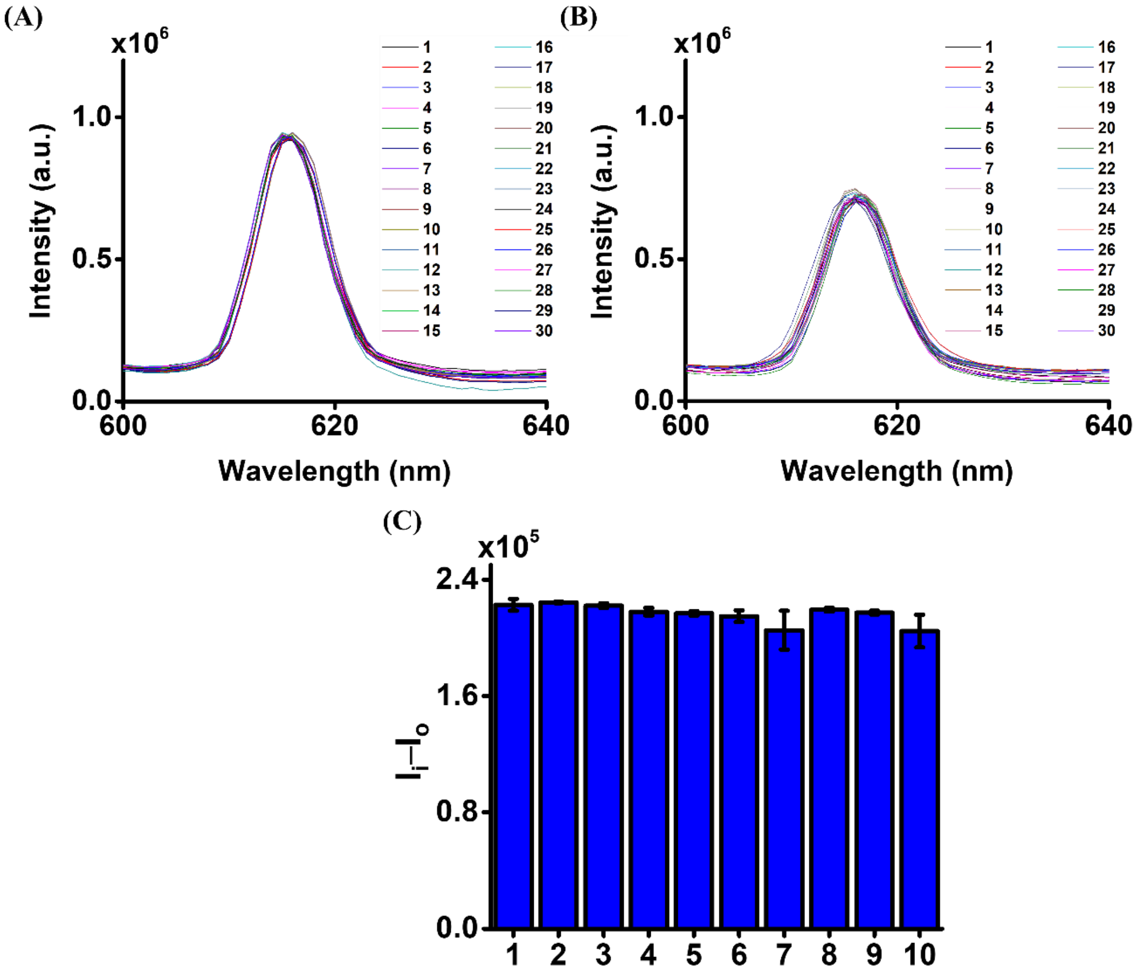

Figure S6. Examination of precision and accuracy. Fluorescence spectra of (A) the sample containing TC (40 nM) and (B) Tris buffer (pH 6, 10 mM) obtained after being treated by our developed method for 30 runs (6 runs per day for 5 days), (C) Bar graphs for summarizing the results of the same sample from 30 runs. .

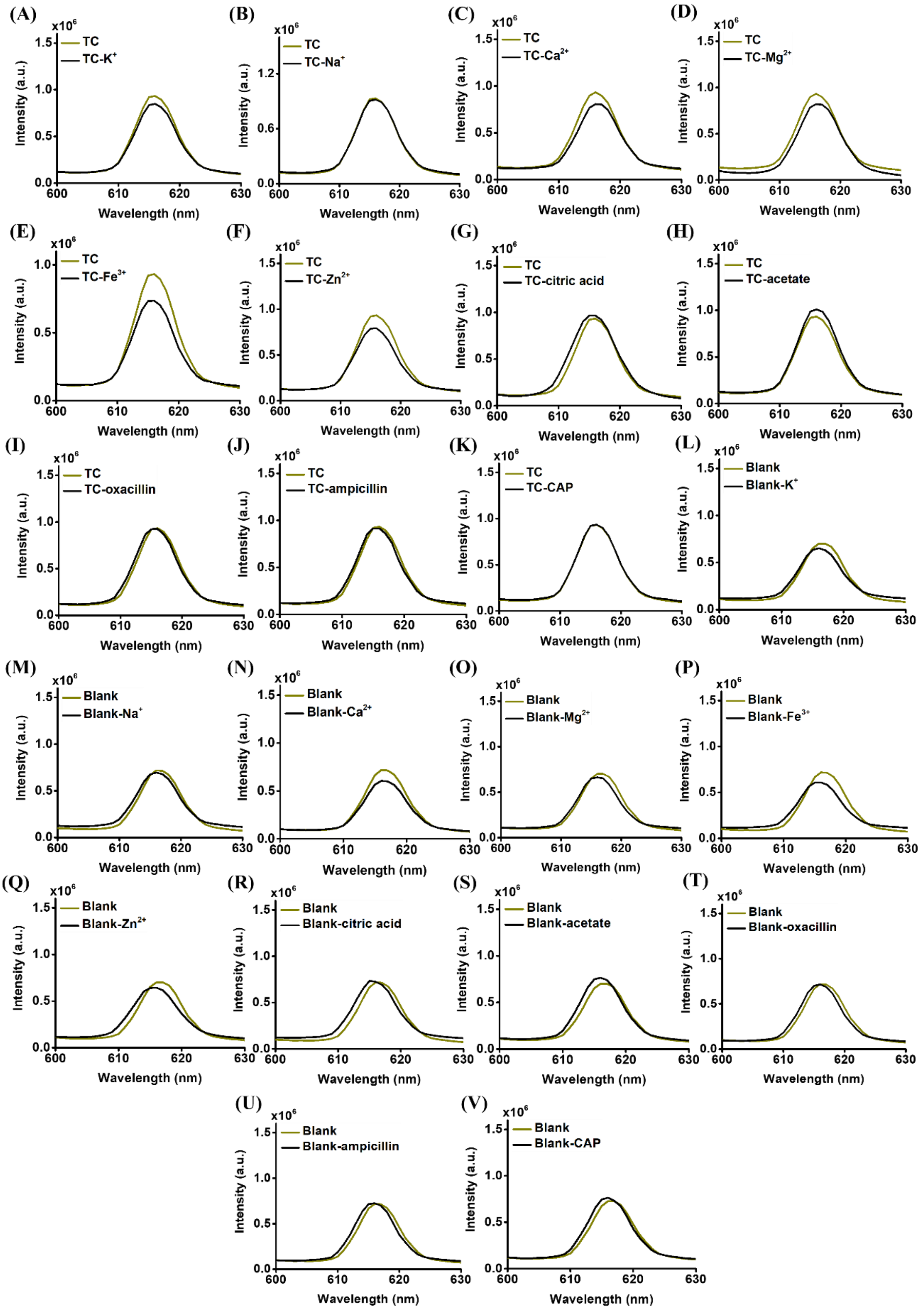

Figure S7. Examination of interference effects and selectivity of the developed method. Fluorescence spectra (λ_{ex}= 394 nm) of the sample (6 mL) containing TC (40 nM) in the absence (green) and presence (black) of interference species (400 nM) including (A) K^+, (B) Na^+, (C) Ca^{2+}, (D) Mg^{2+}, (E) Fe^{3+}, (F) Zn^{2+}, (G) citrate, (H) acetate, (I) oxacillin, (J) ampicillin, and (K) CAP. (L)–(V) are the corresponding blank spectra to (A)–(K), except that TC was not added to these blank samples.

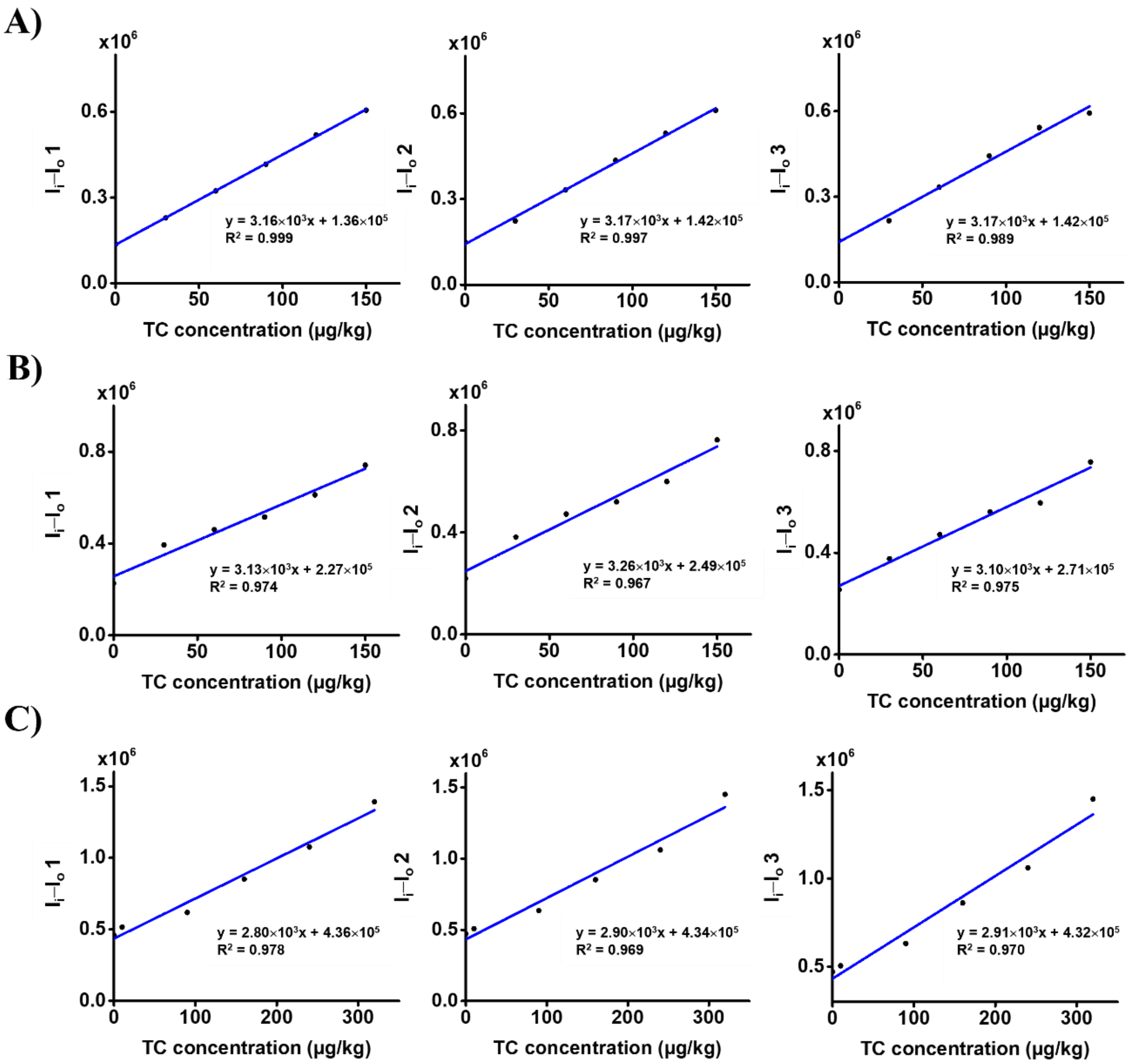

Figure S8. Fluorescence spectra of the chicken broth samples spiked with TC at the concentrations of (A) 50, (B) 100, and (C) 200 μg kg^{-1} obtained using our method.

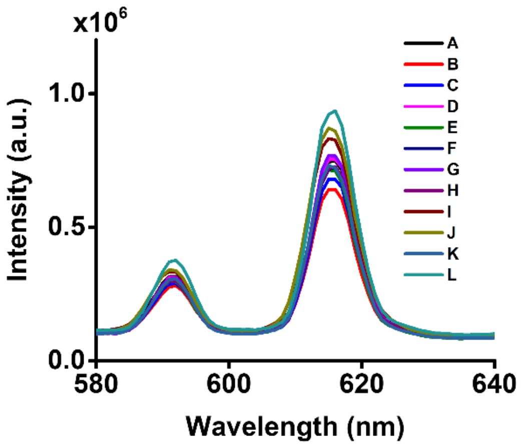

Figure S9. Fluorescence spectra of the blind samples (A-L) obtained after using our method.

References

1. Huy, B. T.; Nghia, N. N.; Lee, Y.-I. Highly sensitive colorimetric paper-based analytical device for the determination of tetracycline using green fluorescent carbon nitride nanoparticles. *Microchem. J.* **2020,** *158,* 105151.
2. Zhang, J.; Shi, G. Rational design of MoS_2 QDs and Eu^{3+} as a ratiometric fluorescent probe for point-of-care visual quantitative detection of tetracycline via smartphone-based portable platform. *Anal. Chim. Acta.* **2022,** *1198,* 339572.
3. Shen, Y.; Wei, Y.; Chen, H.; Wu, Z.; Ye, Y.; Han, D.-M. Liposome-encapsulated aggregation-induced emission fluorogen assisted with portable smartphone for dynamically on-site imaging of residual tetracycline. *Sens. Actuators, B* **2022,** *350,* 130871.
4. Xu, J.; Shen, X.; Jia, L.; Zhou, T.; Ma, T.; Xu, Z.; Cao, J.; Ge, Z.; Bi, N.; Zhu, T. A novel visual ratiometric fluorescent sensing platform for highly sensitive visual detection of tetracyclines by a lanthanide-functionalized palygorskite nanomaterial. *J. Hazard. Mater.* **2018,** *342,* 158-165.
5. Jia, L.; Guo, S.; Xu, J.; Chen, X.; Zhu, T.; Zhao, T. A ratiometric fluorescent nano-probe for rapid and specific detection of tetracycline residues based on a dye-doped functionalized nanoscaled metal–organic framework. *Nanomaterials* **2019,** *9* (7), 976.
6. Han, L.; Fan, Y. Z.; Qing, M.; Liu, S. G.; Yang, Y. Z.; Li, N. B.; Luo, H. Q. Smartphones and test paper-assisted ratiometric fluorescent sensors for semi-quantitative and visual assay of tetracycline based on the target-induced synergistic effect of antenna effect and inner filter effect. *ACS Appl. Mater. Interfaces* **2020,** *12* (41), 47099-47107.
7. Ti, M.; Li, Y.; Li, Z.; Zhao, D.; Wu, L.; Yuan, L.; He, Y. A ratiometric nanoprobe based on carboxylated graphitic carbon nitride nanosheets and Eu^{3+} for the detection of tetracyclines. *Analyst* **2021,** *146* (3), 1065-1073.
8. Jia, L.; Chen, R.; Xu, J.; Zhang, L.; Chen, X.; Bi, N.; Gou, J.; Zhao, T. A stick-like intelligent multicolor nano-sensor for the detection of tetracycline: The integration of nano-clay and carbon dots. *J. Hazard. Mater.* **2021,** *413,* 125296.
9. Zhang, L.; Wang, Y.; Jia, L.; Bi, N.; Bie, H.; Chen, X.; Zhang, C.; Xu, J. Ultrasensitive and visual detection of tetracycline based on dual-recognition units constructed multicolor fluorescent nano-probe. *J. Hazard. Mater.* **2021,** *409,* 124935.
10. Yao, R.; Li, Z.; Liu, G.; Fan, C.; Pu, S. Luminol-Eu-based ratiometric fluorescence probe for highly selective and visual determination of tetracycline. *Talanta* **2021,** *234,* 122612.
11. Wang, T.; Mei, Q.; Tao, Z.; Wu, H.; Zhao, M.; Wang, S.; Liu, Y. A smartphone-integrated ratiometric fluorescence sensing platform for visual and quantitative point-of-care testing of tetracycline. *Biosens. Bioelectron.* **2020,** *148,* 111791.
12. Xu, J.; Guo, S.; Jia, L.; Zhu, T.; Chen, X.; Zhao, T. A smartphone-integrated method for visual detection of tetracycline. *Chem. Eng. J.* **2021,** *416,* 127741.
13. Song, J.; Liu, X.; Zhang, X.; Fan, J.; Zhang, R.; Feng, X. A smartphone-assisted paper-based ratio fluorescent probe for the rapid and on-site detection of tetracycline in food samples. *Talanta* **2023,** *265,* 124874.

https://doi.org/10.3390/bios16010029
